# Accelerated self-gated UTE of murine heart

**DOI:** 10.1186/1532-429X-16-S1-P59

**Published:** 2014-01-16

**Authors:** Wolter L de Graaf, Abdallah Motaal, Nils Noorman, Luc Florack, Klaas Nicolay, Gustav J Strijkers

**Affiliations:** 1 Biomedical NMR, Department of Biomedical Engineering, Eindhoven University of Technology, Eindhoven, Netherlands; 2 Department of Mathematics, Eindhoven University of Technology, Eindhoven, Netherlands

## Background

Sequences with ultra-short echo time (UTE) are popular for their ability to measure tissue components with short T2*. For cardiac applications, UTE MRI was recently employed to detect fibrosis after myocardial infarction [1] and diffuse fibrosis during progression to heart failure [2]. Here we introduce a self-gated UTE MRI sequence for the mouse heart with golden-angle radial acquisition and compressed sensing (CS) reconstruction.

## Methods

**Figure 1:**
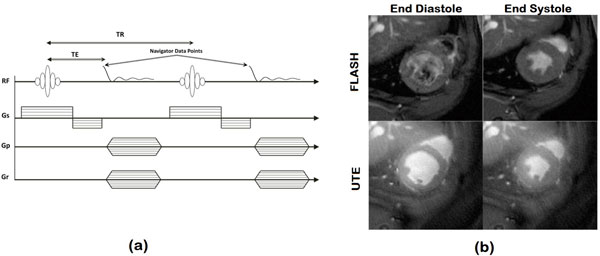
**(a) The self-gated UTE sequence**. (b) Comparison between (top) FLASH and (bottom) self-gated UTE reconstruction at (left) end-diastole and (right) end-systole.

Mice were measured at 9.4T. A self-gated UTE sequence was implemented as shown in Figure 1a. Sequence parameters were: 15o; TR = 9.5 ms; TE = 0.33 ms; field of view = 3 × 3 cm 2; readout data (Number Radial Spokes (NS) × Spoke Length) = 604 × 96; reconstruction matrix = 256 × 256; slice thickness = 1 mm; number of slices = 6; number of repetitions (NR) = 125; number of frames = 15. Navigator analysis was done using home-built Matlab software. Undersampled data, i.e. with shorter acquisition time, were taken from the first 0.7, 1 and 2 min of the data at every slice. Reconstructions of the undersampled datasets, corresponding to 6X, 4X and 2X k-space under-sampling, were done by CS reconstruction. Data was compared to standard self-gated Cartesian FLASH Cine.

## Results

**Figure 2:**
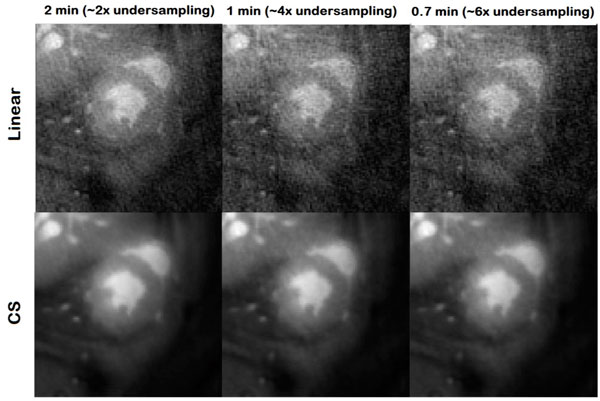
**Comparison between linear and CS reconstructions for 2-, 4-, and 6X undersampling kt space at end-systole**.

Figure 1b shows the difference between the self-gated FLASH and self-gated-UTE of short-axis mid-ventricle views of the heart at end-diastole and end-systole. UTE results in less flow artifacts and saturated blood problems with almost uniform blood intensity in the LV and RV cavities. Figure 2 shows still frames at end-systole for the standard reconstruction, compared with the corresponding 2X-, 4X- and 6X linear and accelerated CS reconstructions. The image quality of the standard acquisition was nearly recovered for the 2X, 4X and 6X CS accelerations. Image quality of movies with higher undersampling factors were visually judged insufficient for evaluation. We therefore limited further analyses to these three accelerated movies. The CS reconstruction recovered good quality images of the heart in which the endocardium and papillary muscles were well distinguishable. To evaluate the effect of CS reconstruction on heart functional parameters (EDV, ESV, EF), standard and 2X, 4X and 6X CS reconstructions were statistically compared in Bland-Altman plots. Very good agreement between accelerated and fully sampled acquisitions was obtained.

## Conclusions

6X undersampled UTE Cine acquisition of the mouse heart was achieved with high image quality and without significantly compromising the determination of cardiac functional parameters. Whole heart coverage can be obtained in less than 8 min.

## Funding

This research is funded by the Imaging Science & Technology group Eindhoven.
